# Membrane-Based Characterization of a Gas Component — A Transient Sensor Theory

**DOI:** 10.3390/s140304599

**Published:** 2014-03-07

**Authors:** Detlef Lazik

**Affiliations:** Helmholtz-Centre for Environmental Research, T.-Lieser-Strasse 4, Halle 06120, Germany; E-Mail: detlef.lazik@ufz.de; Tel.: +49-345-558-5209; Fax: +49-345-558-5559

**Keywords:** sensor, membrane, selectivity, gas, identification, discrimination, quantification

## Abstract

Based on a multi-gas solution-diffusion problem for a dense symmetrical membrane this paper presents a transient theory of a planar, membrane-based sensor cell for measuring gas from both initial conditions: dynamic and thermodynamic equilibrium. Using this theory, the ranges for which previously developed, simpler approaches are valid will be discussed; these approaches are of vital interest for membrane-based gas sensor applications. Finally, a new theoretical approach is introduced to identify varying gas components by arranging sensor cell pairs resulting in a concentration independent gas-specific critical time. Literature data for the N_2_, O_2_, Ar, CH_4_, CO_2_, H_2_ and C_4_H_10_ diffusion coefficients and solubilities for a polydimethylsiloxane membrane were used to simulate gas specific sensor responses. The results demonstrate the influence of (*i*) the operational mode; (*ii*) sensor geometry and (*iii*) gas matrices (air, Ar) on that critical time. Based on the developed theory the case-specific suitable membrane materials can be determined and both operation and design options for these sensors can be optimized for individual applications. The results of mixing experiments for different gases (O_2_, CO_2_) in a gas matrix of air confirmed the theoretical predictions.

## 1. Introduction

Gases such as carbon dioxide (CO_2_), oxygen (O_2_), methane (CH_4_) and hydrogen (H_2_) are important for various environmental and technical processes. There is an increasing need to monitor gases in the environment, for instance within a subsurface, using measurement techniques capable of integrating locally fluctuating gas concentrations from heterogeneous natural systems, such as soils, aquifers and surface water bodies, or geotechnical/technical systems (repositories and reactors). Therefore, robust monitoring systems that (*i*) gather information from large areas; (*ii*) work efficiently within a subsurface; (*iii*) are insensitive to changes in phase saturation and (*iv*) respond rapidly are required.

**Figure 1.**
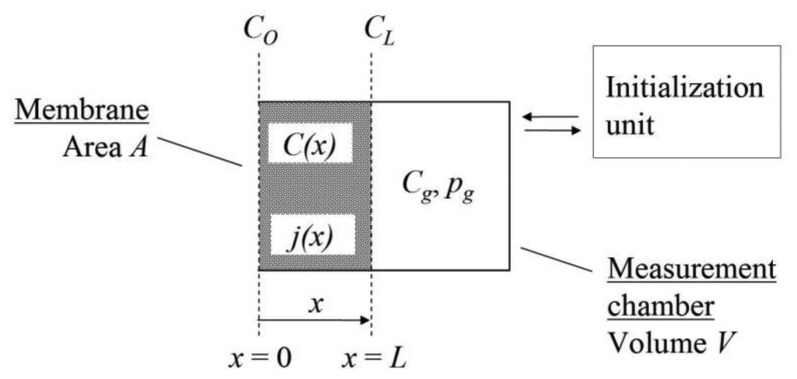
The cell of a membrane-based gas sensor: *C*(*x*), *C_0_*, *C_L_* are the concentrations and *j*(*x*) is the flux density of a gas within the membrane; *C_g_*, *p_g_* are the concentration and gas pressure within the measurement chamber. Initial gas concentrations for the measurement chamber are provided using an initialization unit.

Therefore, a suitable technology, which was first introduced in [1], was developed [2] based on gas diffusion through the wall of a gas-selective membrane into a closed measurement chamber (Figure 1). The individual gas fluxes are superposed on each other and thus change the total mole number within that chamber. This change will vary either the gas volume or the chamber pressure, assuming a closed measurement chamber, which can be related to the partial pressure/concentration of the investigated gas.

The shape of the sensor cell (Figure 1) can be adapted for various measurement problems. For example, tubular membranes can be used to form linear sensor cells (line-sensors) that integrate over a large area and sample a significant range of the locally fluctuating concentrations independent of the supporting area phase, which is advantageous for analyzing gases over large areas. In contrast, gas sensors have been progressively used for automotive engineering, air conditioning, the medical and health industry, numerous laboratory applications and safety systems (fire and gas alarms). Therefore, gas analytical/sensor solutions are often miniaturized. A comprehensive survey of gas sensing technologies for such applications was recently performed in [3].

One advantage of membrane-based gas sensors is their applicability for differing gas components. The sensor must be calibrated for the targeted gas component within a given gas matrix, e.g., air. This interesting feature was successfully used to monitor different mixtures of air and O_2_ or CO_2_ *in situ* within a lysimeter filled with soil [4]. In addition, an installed sensor cell can be calibrated without dismounting under an unknown background concentration [5]. The disadvantage is that choosing the desired calibration requires that the gas component varying in the gas matrix be known. A previous work demonstrated a means of overcoming this disadvantage by solving a system of equations using a set of measurement chambers coated with diverse gas-selective membranes [2]. However, the construction of such a sensor set increases both the technical and maintenance requirements.

A novel sensor approach is introduced in this work to identify and quantify gas components in a given gas matrix. The resulting sensor cell (shortened as “cell” throughout the paper) is robust, simply constructed and applicable to various gases. A suitable gas-selective membrane for such a cell can be selected from a high number of dense polymers, ceramics or metal films. The corresponding material parameters are available from current gas separation research and material data collections [6–9].

## 2. Transient Sensor Theory

### 2.1. Gas Diffusion into a Closed Chamber Coated by a Planar Membrane

According to the solution-diffusion model, a gas molecule permeates through a dense symmetrical membrane in several steps. First, gas from an adjacent space is adsorbed onto the membrane surface. Once a gas molecule is adsorbed, whose desorption or absorption depends on the surface energetics. Absorption, which is a dissolution process, is the rate-limiting step relative to the rapid adsorption process.

Gas molecules diffuse within the membrane according to a concentration gradient. The flux density, *j*(*x*) (mol/m^2^/s), is described by Fick's first law, *j*(*x*) = –*D*·*dC*/*dx* where *D* (m^2^/s) is the gas diffusion coefficient, *C* (mol/m^3^) is the concentration within the membrane, *x* (m) is the distance to the membrane surface and *t* (s) is the time. Assuming a constant diffusion coefficient, for gas movement through a membrane holds the mass balance: *D·d*^2^*C*/*dx*^2^ = *dC*/*dt.*

If a gas molecule reaches the opposite membrane face, mass transfer proceeds in the reverse order: the gas evaporates from the membrane phase and subsequently desorbs into the gas phase.

Because both the adsorption-desorption and gas diffusion processes outside the membrane are rapid relative to diffusion within the solid membrane, an adsorption-desorption equilibrium can be approached in the adjacent gas spaces. For sufficiently low concentrations, a general nonlinear adsorption isotherm can be approximated using a linear partition equilibrium: *C* = *S*·*Cg*, where *S* [-] is the solubility and *Cg* (mol/m^3^) is the concentration within the gas phase.

Assuming, the concentration in the outer membrane face of a cell (according to Figure 1) is given by the boundary condition:

(1) $${C(t)|}_{x=0}=C_{L}+(C_{0}-C_{L})\cdot h(t-t_{0})$$

where *C*_0_ and *C_L_* (mol/m^3^) are the gas concentrations in the outer membrane face at *x* = 0 and inside the chamber at *x* = *L*, respectively, *L* (m) is the membrane thickness, *h* is the Heaviside step function and *t*_0_ (s) is a time constant. For the opposite membrane face within the chamber, the boundary condition should obey:

(2) $${C(t\leq t_{0})|}_{x=L}=C_{L}$$

The following two cases were considered for the initial concentration:

(3) $${C(x)|}_{t=t_{0}}=\{\begin{array}{cc} \left(1-\delta \right)\cdot (C_{0}-C_{L})+C_{L}, & \left(I\right)\\ C_{L}, & \left(II\right) \end{array}$$

where *δ* = *x*/*L* is a dimensionless distance. Case (I) defines a dynamic equilibrium resulting in a steady-state flow of gas into the chamber. Case (II) defines the thermodynamic equilibrium (partition equilibrium) for concentrations within and outside the membrane.

The flux density at the inner membrane face (area A (m^2^)) into the closed chamber (volume V (m^3^)) is as follows:

(4) $${j(t)|}_{x=L}=\frac{L}{\vartheta }\cdot \frac{\partial C(t)}{\partial t}=\frac{V}{A}\cdot \frac{\partial Cg(t)}{\partial t}$$

where *ϑ* = *ν*/*ν_g_* = *S·AL*/*V* is the dimensionless ratio of the total mole numbers for the gas within the membrane (*_v_* (mol)) and chamber (*ν_g_* (mol)) in the equilibrated system.

Applying the Laplace transformation method to that problem an analytical solution can be constructed for the normalized concentration *C̃* via the semi-infinite series:

(5) $$\frac{C-C_{L}}{C_{0}-C_{L}}=\tilde{C}(\delta ,\tau )=1-\sum_{k=1}^{\infty }\frac{2\left(1-\phi +\phi \cdot \sin\text{c}\;\lambda_{k}\right)\sin\text{c}\;\lambda_{k}}{1+\sin\text{c}\;\lambda_{k}\cos\lambda_{k}}\cdot \frac{\sin\lambda_{k}\delta }{\sin\lambda_{k}}\cdot \exp(-\lambda_{k}^{2}\tau )$$

Where *τ* = *D*(*t*−*t*_0_)*L*^-2^ is a dimensionless time parameter. The parameter *ϕ* defines the particular solution with respect to the initial gas concentration according to Equation (3). For case (I), the dynamic equilibrium condition requires *ϕ* = 1, while for case (II), the thermodynamic equilibrium condition holds at *ϕ* = 0. The eigenvalue, *λ_n_*, is the nonzero positive root of *λ_k_* · tan *λ_k_* = *ϑ*.

Equivalent analytical solutions of corresponding heat conduction problems are presented, e.g., in [10,11] and discussed in detail. For example, [11] details a slab of length *l*, density *ρ*, specific heat capacity *c* and heat conductivity *K* in contact with one face with a well-stirred fluid with a mass per unit area of *M′* and specific heat capacity of *c′* (see Chapter 3.1.9, pp. 128–129). Here, the ratio *ρc*/(*M′*/*l*·*c′*) and thermometric conductivity *κ* = *K*/(*ρc*) appear instead of the solubility *S* and diffusion coefficient *D*, respectively.

### 2.2. Pressure Evolution in the Measurement Chamber

Because the adsorption/desorption processes are comparatively fast, the concentration within the membrane surface is approximately in equilibrium with the concentration in the adjacent gaseous phase, which can be described using its dimensionless pressure, *p* = *RT*·*Cg*/*p_n_* (where *p_n_* (Pa) is the absolute gas pressure, R = 8.314 J/mol/K is the gas constant and T (K) is the temperature).

If different gas components (index “*j*”) constitute the gaseous phase the pressure is determined by the sum of these partial pressures *p^j^* according to Dalton's law. Furthermore, if the superposition principle holds for the solution and diffusion of individual gas components within the membrane, the pressure, *p*, within the measurement chamber follows from Equation (5) with:

(6) $$\begin{array}{l} p=\sum_{j}p^{j}=\underset{j}{\text{∑}}p_{L}^{j}+(p_{0}^{j}-p_{L}^{j})\left(1-\overset{n\to \infty }{\underset{k=1}{\text{∑}}}\beta_{k}^{j}(\phi )\cdot \exp(-\lambda_{k}^{j^{2}}\alpha_{j,s}\tau_{s})\right)\\ \beta_{n}^{j}(\phi )=\frac{2\left(1-\phi +\phi \sin\text{c}\;\lambda_{k}^{j}\right)\sin\text{c}\;\lambda_{k}^{j}}{1+\sin\text{c}\lambda_{k}^{j}\cos\lambda_{k}^{j}} \end{array}$$

where the dimensionless time *τ_s_* = *D_s_*(*t*−*t*_0_)*L*^-2^ and relative diffusivity *α_i,s_* = *D_i_* / *D_s_* of gas component “*j*” are defined with respect to the gas component “*s*”. The dimensionless partial pressures $p_{0}^{j}$, $p_{L}^{j}$ are related to the respective initial concentrations $C_{0}^{j}$, $C_{L}^{j}$ of gas component “*j*”. The eigenvalues in Equation (6) must be separately determined from $\lambda_{k}^{j}\tan\lambda_{k}^{j}=\vartheta_{j}=S_{j}\cdot AL/V$ for each gas component.

Equation (6) represents the exact pressure evolution within a measurement chamber for two operating modes: case (I) begins from an established dynamic equilibrium in which the maximum gas flow passes through the gas selective membrane, and case (II) starts from thermodynamic equilibrium (no gas flow).

#### Approximations

In general, as demonstrated in Section 4, the Equation (6) cannot be examined as a simple gas-specific sum of exponential equations. Nevertheless, for eigenvalues $\lambda_{1}^{j}\ll 1$ e.g., for a comparatively thin membrane (*AL*/*V* ≪ 1), the terms $\lambda_{1}^{j}\cdot \tan\lambda_{1}^{j}=\vartheta_{j}$ can be approximated by ${\left(\lambda_{1}^{j}\right)}^{2}\approx \vartheta_{j}=S_{j}\cdot AL/V$. Because the first-order coefficients inside the exact solution in case (I) are near $\beta_{1}^{j}(\phi =1)\approx 1$ and by disregarding the small higher-order coefficients, Equation (6) can be simplified as follows:

(7) $$p=\sum_{j}p_{L}^{j}+(p_{0}^{j}-p_{L}^{j})\cdot \left(1-\exp(-(t-t_{0})gf_{j,s}P_{s})\right)$$

where the sensor geometry is *g* = *A*/*V L*, the permeation selectivity (also called the permselectivity or selectivity) of gas component “*j*” with respect to gas component “*s*” is *f_i,s_* = *P_j_*/*P_s_* and *P_i_* = *S_i_ D_i_* is the permeability of the membrane for gas component “*i*”. Equation (7) corresponds to the thin-layer solution presented in [2]. For short times, Equation (7) yields the following linearized term:

(8) $$a_{1}^{\left(I\right)}={\frac{dp}{dt}|}_{t\to +t_{0}}=gP_{s}\sum_{j}f_{j,s}(p_{0}^{j}-p_{L}^{j})$$

where the pressure change, *dp*/*dt*, within the measurement chamber relates for the case (I) to the partial pressure differences between the faces of the membrane. This relation was successfully used to both simultaneously estimate O_2_ and N_2_ concentrations using two measurement chambers covered with membranes of differing selectivities [2] and analyze a single gas, such as O_2_ or CO_2_, mixed in air [4] using only a single membrane-based gas sensor (one cell). The notation “*a*_1_” originates from the measurement procedure based on approximating the recorded pressure evolution using a polynomial, *p* = *a*_0_ + *a*_1_(*t*–*t*_0_) + *a*_2_(*t*–*t*_0_)^2^ + … Disregarding the higher-order terms for short times, *t* – *t*_0_, and differentiating yields *dp*/*dt* = *a*_1_.

However, if *t* ≫ *t*_0_, the first terms (*k* = 1) of the summation dominate, and Equation (6) develops exponentially with time:

(9) $$p=\sum_{j}p_{L}^{j}+(p_{0}^{j}-p_{L}^{j})\cdot \left(1-\beta_{1}^{j}(\phi )\cdot \exp(-\lambda_{1}^{j^{2}}\alpha_{j,s}\tau_{s})\right)$$

Hence, with the restriction of $\lambda_{1}^{j}\ll 1$ (see above), Equation (7) should be approximately valid for both cases (I) and (II) in this long-term range.

### 2.3. Determination of a Gas Component

A concentration-independent criterion is necessary to separate information on the gas component that changes the composition of a given gas matrix. To this end, Equation (5) will be examined. Here, the absolute concentration, *C*, is defined by *C_L_* and (*C_0_* – *C_L_*), whereas, independent of the absolute concentration, its time response (evolution) depends only on parameters *D_i_* and *S_i_*, which describe the material properties with respect to a specific gas component “*i*”, and parameters *AL*/*V* and *L*, which describe the gas sensor geometry. The pressure evolution based on Equation (6), which results from the superposition of Equation (5) for different gas components, behaves in an analogous way. Thus, normalizing of the pressure evolution within a first measurement chamber (pressure *p*_1_) by that of a second measurement chamber (pressure *p*_2_) with different geometrical and/or material parameters should result in a characteristic function *f*(*t*|*L*_1_,*L*_2_,*A*_1_,*A*_2_,…). This function must be independent of the concentration but should be characteristic for the mixed gas component as long as its diffusion coefficient or solubility differs from the corresponding coefficients of the other gas components. Therefore, rearranging Equation (6) for the pressure evolutions $p^{\prime }=p_{1}-\sum p_{L}^{j}$ and $p^{\prime \prime }=p_{2}-\sum p_{L}^{j}$ yields the following:

(10) $$\frac{p^{\prime }}{p^{\prime \prime }}=f(t|L_{1},L_{2},A_{1},A_{2},\ldots )=\frac{\underset{j}{\text{∑}}(p_{0}^{j}-p_{L}^{j})\overset{n\to \infty }{\underset{k=1}{\text{∑}}}{\beta^{\prime }}_{k}^{j}(\phi )\cdot \exp(-{\lambda^{\prime }}_{k}^{j^{2}}{\alpha^{\prime }}_{j,s}{\tau^{\prime }}_{s})}{\underset{j}{\text{∑}}(p_{0}^{j}-p_{L}^{j})\overset{n\to \infty }{\underset{k=1}{\text{∑}}}{\beta^{\prime \prime }}_{k}^{j}(\phi )\cdot \exp(-{\lambda^{\prime \prime }}_{k}^{j^{2}}{\alpha^{\prime \prime }}_{j,s}{\tau^{\prime \prime }}_{s})}(10)$$

where all of the coefficients and parameters must be enumerated with respect to the individual cells. Approximate terms are specified in the same manner using Equations (9) and (7).

Assuming the function *f*(*t*) = *p′*/*p″* behaves monotonically, and within the range of interest holds {*p′*, *p″*} ≠ 0, then a specific point of intersection with a suitable separating function *γ*:

(11) $$p^{\prime }/p^{\prime \prime }-\gamma =0$$

exists at the critical time $t_{c}^{i}$. This critical time will be related to a particular gas component “*i*” that is mixed or varied in the gas matrix. The separating function *γ* can be a constant or a function of time. The case, in which *γ* = 1, is of special importance for measurement practices. Considering that the common denominator *p″* does not change the time response, it can be neglected in Equation (11). Thus, a simple pressure difference is sufficient to determine $t_{c}^{i}$:

(12) $$\Delta p(t)=p^{\prime }-p^{\prime \prime }$$

**Figure 2.**
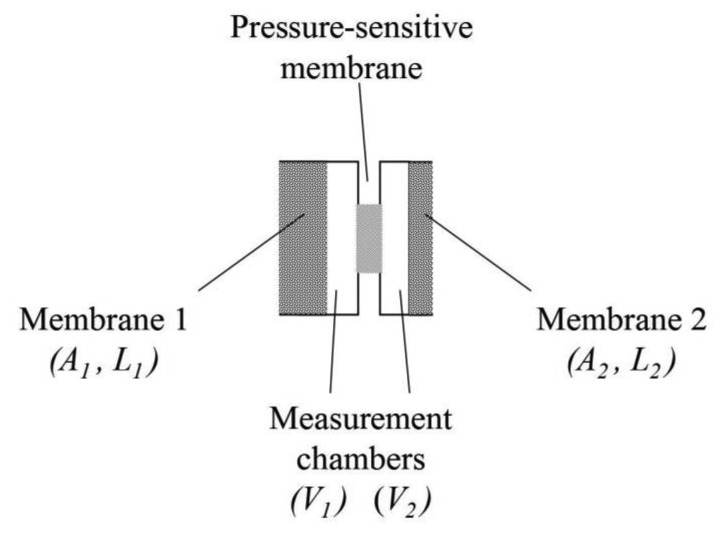
Combination of two cells enables a gas component to be indentified and its concentration quantified for case (II). The extension of the paired cells by an initialization unit to adjust steady-state flow condition enables use with case (I).

This pressure difference can be analyzed using only a single differential pressure sensor according to Figure 2, which has great technological significance. Here, the critical time is marked by the temporal distance between a pressure sensor agitation and the subsequent pressure signal intersection with the sensor baseline Δ*p* = 0 or a time-dependent baseline determined using a reference measurement.

Furthermore, the differential pressure Δ*p*(*t*) of an agitated pressure sensor for short times is primarily determined by *p′* or *p″* depending on the parameterization. Therefore, the differential pressure change d(Δ*p*)/*dt* for short times enables gas concentration quantification.

For case (I), the quantification is based on applying Equation (8) using previously described measurement technology (for details, see [4]). In contrast to case (I), which assumes dynamic equilibrium (steady-state flows for all gases), case (II) considers a sudden change in the gas composition at the outer membrane face at *t* = *t*_0_. Hence, a time lag for gas diffusion through the membrane walls, *t_L_*, must be considered, and the sensor response is time-shifted. Such a case will be considered in Section 4.

## 3. Computational Simulations and Experiments

### 3.1. Computational Simulations

Varying geometrical sensor properties were considered for computational simulations (codes written in Mathematica 7, Wolfram Research, Oxfordshire, UK) using the dense membrane material dimethylsiloxane (plus 33% silica filler by weight), resulting in the simplifications: *α′* = *α″* = *α* and *τ″* = (*L*_1_/*L*_2_)^2^ · *τ′*, in Equation (10) and the corresponding approximated relations. The relevant membrane parameters are shown in Table 1 (data from [9], p. 424, the first table) which contains data from [12].

Tipping errors in [9], which were identified in comparison to the original paper [12] are revised in Table 1. In detail, the same numerical values originally reported by Robb [12] in the solubility unit “cm^3^(RTP)/(cm^3^ × atm)” are given in [9] with a different unit, “ml/g” and the displayed diffusion coefficients are one order of magnitude too high in [9].

The permeabilities shown in Table 1 were calculated to guarantee comparable data sets for the comparison of exact and approximate solutions and were thus not obtained from independent experiments.

Three configurations were investigated for a cell according to Figure 1. Cell I was parameterized with a membrane volume *AL* = 0.1 cm^3^ and a measurement chamber volume *V* = 1 cm^3^, which represents a small ratio of *AL*/*V* ≪ 1. For the same chamber volume *V* = 1 cm^3^, a membrane volume of *AL* = 1 cm^3^ was assumed for cell II. For *AL*/*V* ≫ 1, cell III with a chamber volume of *V* = 0.1 cm^3^ and a membrane volume of *AL* = 1 cm^3^ was simulated. A uniform membrane area *A* = 10 cm^2^ was assumed for all cells. Air was used as the gas matrix for the computational simulations, consisting of 78.09% N_2_, 20.95% O_2_, 0.93% Ar and 0.035% CO_2_. Further simulations were performed using an argon gas matrix, which is used as a cover gas during lamp production, for example.

### 3.2. Experimental

To confirm the theoretical considerations, experiments were performed, in which the measurement starts from the steady-state flow, *i.e.*, from the dynamic equilibrium, case (I). Therefore, a setup consisting of a column (length: 21 cm, inner diameter: 11 cm) containing the membrane-based gas sensor pair was installed in the laboratory and could be flushed with different gas mixtures. Two mass flow controllers (MFC 8712, Bürkert Fluid Control Systems, Ingelfingen, Germany) were used to mix the gas: the first for compressed dry air, and the second added a gas component (CO_2_, O_2_). Reference gauges for O_2_ (a fiber-optic oxygen meter, Fibox 2, PreSens—Precision Sensing, Regensburg, Germany) and CO_2_ (a near-infrared instrument, BCP-CO2, www.getsens.com) were installed in the line connecting the MFCs to the column.

Because planar cells are not yet available, the membrane-based gas sensor pair was constructed using dense tubular membranes (polydimethylsiloxane—PDMS) 4.38 m and 4.40 m in length with 1 mm and 1.1 mm inner diameters, respectively, and 1.9 mm and 2.8 mm outer diameters, respectively. The membranes were installed within the column and were connected with valves (Flipper Solenoid Valve 6124, Bürkert Fluid Control Systems) and a pressure sensor (PCLA12X5D1, Sensortechnics, Puchheim, Germany). The valves and pressure sensor were placed outside at the column cap. Small fittings and short tubes (length: approximately 10 to 15 cm, inner diameter: 1.5 mm, material: PDMS) were integrated in the column cap to connect the valves, and additional tubes with similar lengths and 0.76 mm inner diameters to connect the pressure sensor.

The tubular membranes were flushed at room temperature with dry air from the compressor to adjust the dynamic equilibrium. At the same time, the column was flushed with the adjusted gas mixture. The mean difference between the pressures within the tubular measurement chambers and the gas-flushed column was adjusted to approximately −7 mbar. After establishing a dynamic equilibrium, the measurement chambers were closed by means of the valves, and the pressure evolution between the tubular chambers was recorded.

## 4. Results and Discussion

### 4.1. Comparison of Exact Solutions and Approximations

Tables 2, 3 and 4 contain the first two eigenvalues and corresponding coefficients *β* for the particular solutions Equation (6), cases (I) and (II) for simulated cells I through III. As the tables show, the first coefficient, $\beta_{k=1}^{j}$, dominated over the higher-order coefficients. This tendency increased with decreasing *γ_j_* = *S_j_* · *AL*/*V* and thus with decreasing membrane wall thickness, *L*, or ratio *L*/(*V*/*A*), where *V*/*A* is the mean chamber height. Therefore, if two membranes from the same material but with different wall thicknesses were combined within a sensor pair according to Figure 2, the thicker membrane wall determines the approximation quality to the exact solution.

In addition, the approximation quality depends on the particular solution and time. Tables 2, 3 and 4 demonstrate a significant difference $\beta_{i}^{j}(\phi =1)/\beta_{i}^{j}(\phi =0)\ll 1,(i>1)$ which enhances the computational effort and reduces the approximation quality, notably for short times, for the particular solution case (II) in Equations (6) and (10). Section 4 demonstrates this behavior using Equation (10) as an example.

**Figure 3.**
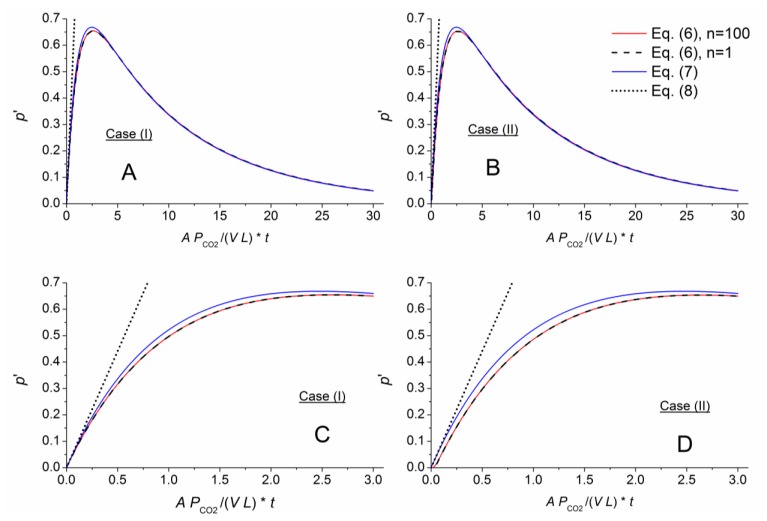
Comparison of the exact solution (Equation (6), n = 100) for *AL*/*V* = 0.1 with the approximations: Equation (6) for *n* = 1, the thin-layer solution Equation (7), and its linear approximation Equation (8). The long-term pressure development occurring after the outer air matrix is replaced with CO_2_ is shown above. A plot for short times is shown below.

**Figure 4.**
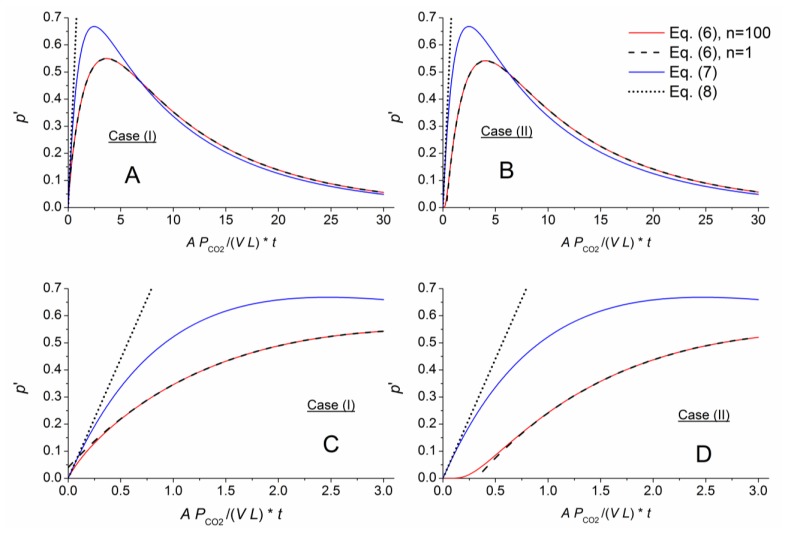
Comparison of the exact and approximate solutions for *AL*/*V* = 1 (same scenario described for Figure 3).

**Figure 5.**
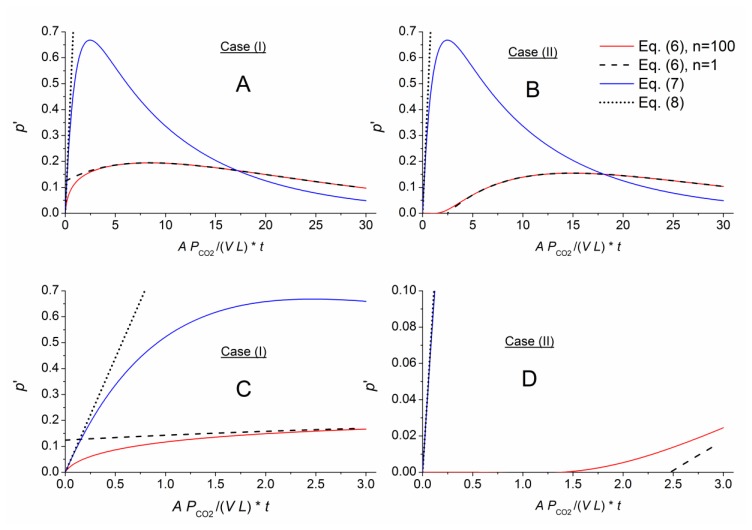
Comparison of the exact and approximate solutions for *AL*/*V* = 10 (same scenario described for Figure 3; note that the scaling in D differs from that of the other plots).

Figures 3, 4 and 5 numerically compare the exact and approximate solutions Equations (6–9) for an exchange of the outer air matrix by CO_2_. Shown are the pressure evolutions $p^{\prime }=p-\sum p_{L}^{i}$. The red lines represent the highly approximated exact solution (sum with *n* = 100 in Equation (6)). The black dashed lines represent Equation (9), being formed by the first term within the sum of Equation (6). The thin-layer solution, Equation (7), is displayed using blue lines, and its linear approximation, Equation (8), is shown as dotted straight lines. To better compare the different cells, the time axes were normalized according to the function argument of the exponential term in Equation (7) with respect to the individual geometry, *g* = *A*/(*V L*), and permeability, *P_CO_*_2_, of the considered gas component, CO_2_.

The different approximate solutions perfectly matched the exact results for the thin membrane. In addition, the thin-layer solution derived from case (I) approximates the exact solution for both cases (I) and (II), and its linearized expression, Equation (8), matched the other solution slopes for short times.

The approximation capability changed for a comparatively thick membrane with *AL* = *L* (Figure 4). Here, the different approximations for Equation (6) only agreed for longer times. Figure 4D shows a slight shift in time of the graphs calculating by the exact (*n* = 100) and approximate (*n* = 1) representations of Equation (6) for case (II). This shift or time lag indicates the necessary time for gas permeation from the outer membrane face through the membrane into the measurement chamber. For a given gas matrix, this lag depends on the gas component, membrane material and sensor geometry. A comparison between Figure 3D and Figures 4D, 5D show a nearly linear relation between the time lag and the *AL*/*V* ratio.

For the short-term section, Equation (7) correlates with Equation (6) in case (I) for *AL* = *V*. Therefore, the approximate solution can be assumed to characterize a gas component at higher *AL*/*V* ratios (short-term and long-term behavior), and Equation (8) can be applied to quantify the concentration. At the same time, the calibration constants needed for measurement gradually lose their physical relationship as the ratio increases and become arbitrary.

Figure 5 shows considerable damping for the exact solution with a cell geometry of *AL* ≫ *V*. Equation (7) fails to describe the pressure in this situation. Therefore, geometries such as those for cell III seem to be unsuitable for sensor applications.

While the short-term results cannot be described by the Equation (9), the long-term behavior was perfectly approximated across all simulations.

**Figure 6.**
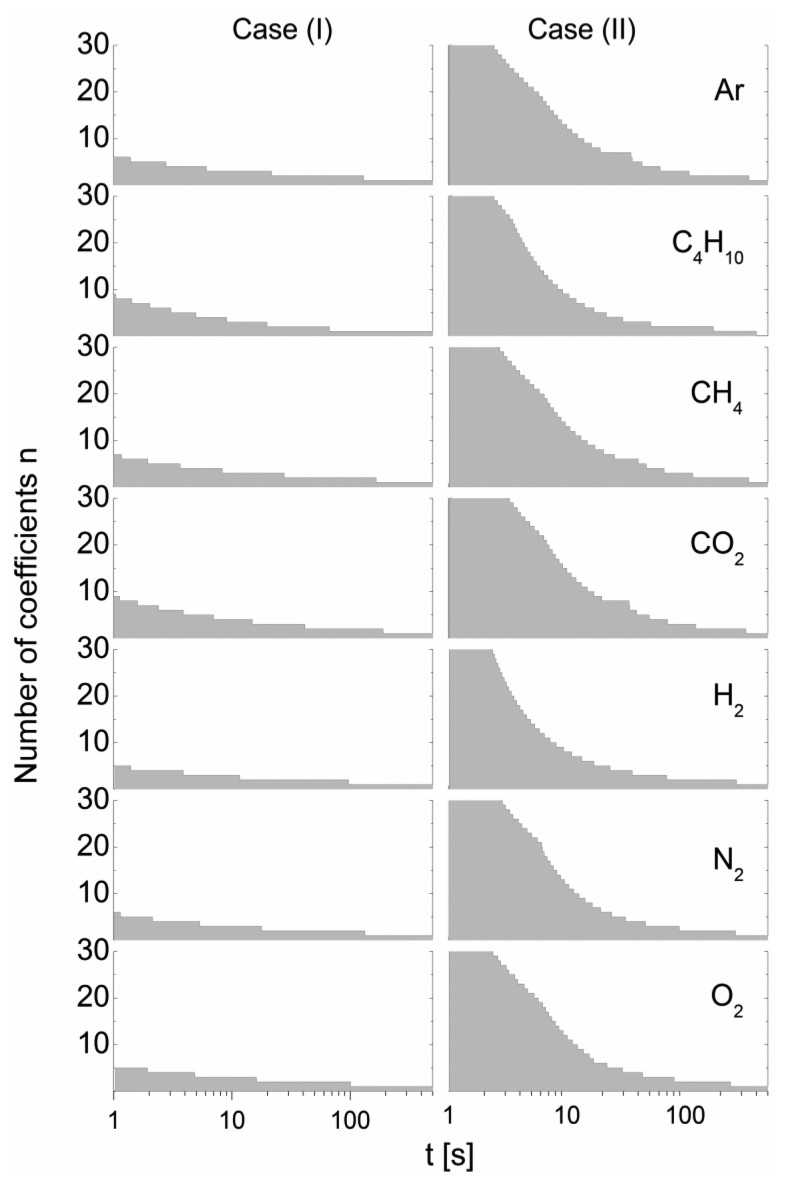
Time dependency of the number of terms *n* in Equation (10) necessary to satisfy the quality criterion *ε* = 0.01 in Equation (13). The gas sensor pair consisted of cell I and cell II.

To highlight the time-dependent approximation behavior of Equation (10), Figure 6 shows the necessary order n, required for the summation to obtain an approximation quality better than *ε* in terms of the relative distance, *p′*/*p″* = *f*(*t*|*n*) from the highly approximated *p′*/*p″* = *f*(*t*|*n_ref_*) exact solution summed over *n_ref_* = 100 terms:

(13) $$\left|\frac{f(t|n)}{f(t|n_{\text{ref}})}-1\right|\leq \varepsilon$$

This simulation was performed for a gas sensor pair consisting of cell I and cell II, with the material parameters outlined in Table 1 and air as the gas matrix, for a relative error of *ε* = 0.01.

Figure 6 shows that a relatively high computational effort is required to achieve the same approximation quality for case (II) as obtained for case (I). The necessary terms exceed *n* > 50 for short times. In addition, due to the differences in material parameters, there arises a significant dependency on the gas added. The general trend of the decrease in necessary terms with increasing time results from the increase in the eigenvalues with increasing *k* according to $\lambda_{k}^{j}/\pi >k-1$, which causes considerable decay of the exponential term.

### 4.2. Characterization of a Gas Component

**Figure 7.**
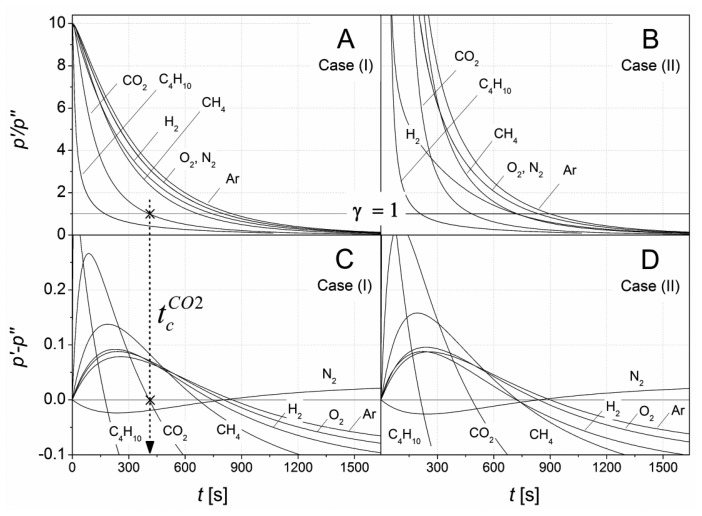
Ratio of *p′*/*p″* simulated for a gas sensor pair formed from cell I and cell II and a outer air gas matrix changing by the marked gas component. The individual graphs for *p′*/*p″* in A and B were transformed into differential pressures in C and D using the separating function *γ* = 1. The arrow marks the critical time, $t_{c}^{CO2}$, for identifying CO_2_.

Figure 7 shows *p′*/*p″* ratios calculated according to Equation (10) for *n* = 100 terms for cases (I) and (II). The gas sensor pair formed from cell I and cell II was simulated by adding an individual gas component to the outer air gas matrix accounting for up to 50% of the final content.

Figure 7A,B shows individual intersections of the separating function γ with the *p′*/*p″* graphs, which enables identification of the added gas component. For *γ* = 1, Equation (11) can be transformed into Equation (12). The resulting differential pressures are shown in Figure 7C,D. The roots for *p′*/*p″* − 1 from Equation (11) and *p′* − *p″* from Equation (12) represent identical critical times.

In addition, the differential pressure according to Equation (12), shown in Figure 7C,D, contains information on the concentration for the identified gas component. For quantification this concentration dependency can be related to the differential pressure change using a gas component specific calibration.

**Figure 8.**
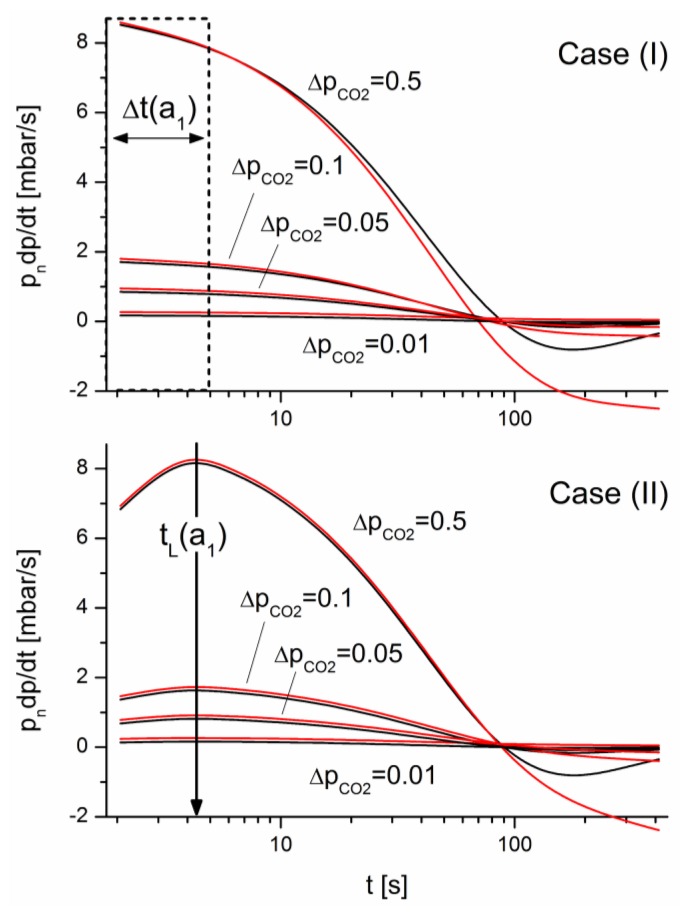
Comparison of derivations *dp*/*dt* (black) for the pressure for cell I and *d*(Δ*p*)/*dt* (red) for the differential pressure for paired cells I and II for differing amounts Δ*p_CO_*_2_ added to air. The calculations consider *n* = 100 terms. To improve the visualization, a vertical offset of 0.1 mbar/s was added to the red lines. The deviations behaved nearly identically within the displayed region Δ*t*(*a*_1_) and near *t_L_*(*a*_1_) necessary for the quantification of a gas component.

The differential pressure changes, *d*(Δ*p*)/*dt*, from Equation (12) for differing CO_2_ concentrations in the air matrix are displayed red in Figure 8 in mbar/s. In comparison, the corresponding signals of the deviation of Equation (6) for the faster cell I which dominates the pressure evolution in Equation (12) for short times are displayed in mbar/s as black lines. To visualize both lines, the red lines were shifted vertically at an amount of 0.1 mbar/s. Furthermore, as shown in Figure 8 for case (II), an extreme pressure change $a_{1}^{\left(II\right)}$

(14) $$a_{1}^{\left(II\right)}={\frac{dp}{dt}|}_{t\to t_{L}}=gP_{s}\sum_{j}f_{j,s}(p_{0}^{j}-p_{L}^{j})$$

occurs independent of the concentration after a time lag, *t_L_*. Analogous to the maximum pressure change in case (I), this extreme value for case (II) is a unique measure to determine the concentration and allow whose calibration. A detailed examination will be given in a separate paper.

**Figure 9.**
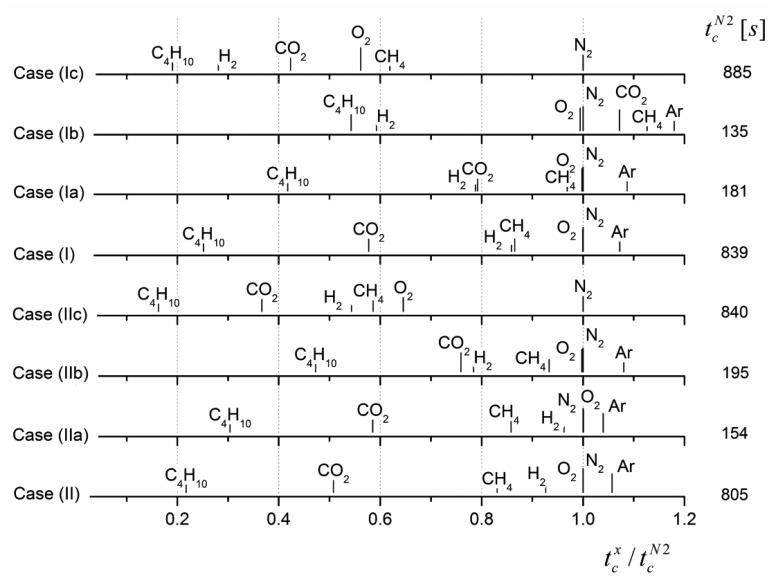
Critical times for the identification of the indicated added gas component depending on: the operating mode, cases (I) and (II); cell configuration; and composition of gas matrix. The simulation parameters are listed in Table 5. The critical times of N_2_ ( $t_{c}^{N2}$) were used to normalize the case-dependent time bases. Corresponding absolute $t_{c}^{N2}$ values are displayed on the right.

Figure 9 shows the final theoretical results for identifying the gas components considered in Table 1. The gas-specific critical times were calculated for different gas matrix compositions for the paired cells I and II. In addition, two special cases were considered with cell configurations that support a further simplification of the respective equations. The parameters for simulation are summarized in Table 5.

The tests for case (I, II) and case (Ic, IIc) represent the cell combination using *A*_1_*L*_1_/*V*_1_ = 0.1 and *A*_2_*L*_2_/*V*_2_ = 1, respectively, considered throughout the paper. Differing solubilities and diffusion coefficients contribute to the pressure difference evolution, depending on the individual cell geometry. Due to the high dilution volumes in cell I comparatively high critical times (right in Figure 9) were observed. A remarkable displacement of critical times occurred upon a change in the gas matrix.

The test case (Ia, IIa) assumed self-similar cell geometries: *A*_1_*L*_1_/*V*_1_ = *A*_2_*L*_2_/*V*_2_, resulting in the independence of the argument *ϑ_j_* = *S_j_* · *AL*/*V* from the individual cells. Assuming self-similarity, Equation (12) can be simplified as follows:

(15) $$\Delta p=\sum_{j}(p_{0}^{j}-p_{L}^{j})\sum_{k=1}^{n\to \infty }{\beta^{\prime }}_{k}^{j}(\phi )\cdot \left(\exp(-{\lambda^{\prime }}_{k}^{j^{2}}\alpha_{j,s}{\tau^{\prime }}_{s})-\exp(-{\left(\frac{L_{1}}{L_{2}}{\lambda^{\prime }}_{k}^{j}\right)}^{2}\alpha_{j,s}{\tau^{\prime }}_{s})\right).$$

For a given gas matrix, the differential pressure Δ*p* is controlled by the wall thickness ratio *L*_1_/*L*_2_, which results in a critical time that scales with the specific gas component time differences for diffusion $(L_{2}^{2}-L_{1}^{2})/D_{j}$. In contrast, *L*_1_ = *L*_2_ can be required and yields a fixed set of diffusion times. In this case the evolution of Δ*p* is determined by differences between the gas solubilities. The tests Ib and IIb shows the simulation results for this case.

Depending on the particular geometry, Figure 9 shows considerable differences in the individual gas component critical times as well as changes in the distance among the critical times of different gas components. Thus, the paired cells can be optimized to discriminate between specific gas components over a wide range. To that aim, the geometrical configuration of individual cells can be varied.

Dependent of the added gas component, the critical time determined using the approximate solution Equation (7), results in relative errors from 0.003 to 1.1 with respect to the exact results. This error is related to the solubility of that gas component. High solubilities cause high eigenvalues (see Tables 2 and 3) and thus, limit the applicability of Equation (7) for the given cell geometries. With a mean relative uncertainty over all simulations of only 0.37, Equation (7) failed to discriminate between different gas components based on the material and geometry parameters.

The error that arises from applying the approximate solution Equation (9) to Equation (12) yields relatively small uncertainties. The relative errors are lower than 0.13, and partly, identical estimations were found with respect to the exact results. The mean relative uncertainty over all simulations was estimated to 0.013. Thus, the approximation seems to be convenient for identifying particular gas components and for adapting the sensor design to specific measurement problems.

### 4.3. Experimental Validation

**Figure 10.**
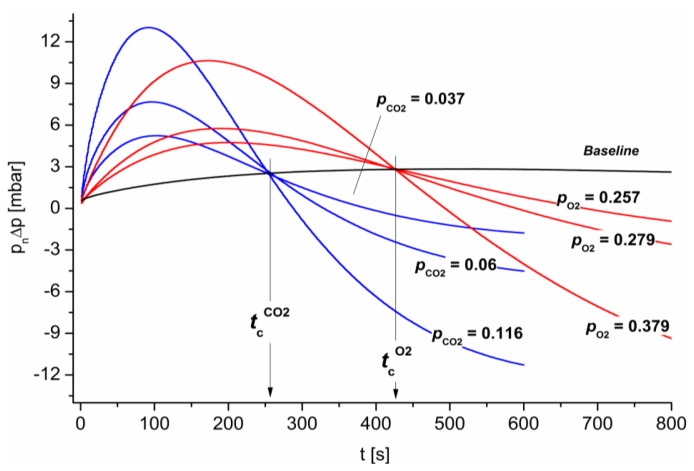
Experimental validation: Gas-dependent evolution of the differential pressure. CO_2_ and O_2_ were added to the air gas matrix in various quantities. The resultant contents are indicated. The individual sensor responses intersect the baseline at a gas-specific critical time, independent of the concentration.

Figure 10 shows validation experimental results representing the operating conditions for case (I). Due to the negative pressure difference (see Section 3.2) between the gas-selective membrane faces, small gas fluxes occur in the tubular measurement chambers, even for equivalent gas compositions within the chambers and column. To compensate for this experimental effect, the resultant differential pressure between both measurement chambers was first determined via an independent experiment. This value defines the baseline in Figure 10. In further experiments, CO_2_ and O_2_ were added in various quantities to the air gas matrix within the column. The resultant contents were measured using reference gauges and are indicated in Figure 10.

As shown in Figure 10, all of the sensor responses intersected the baseline independent of their concentration at a gas-specific critical time ( $t_{c}^{O2}$ or $t_{c}^{CO2}$). In addition, Figure 10 shows a clear correlation between the differential pressure evolution or change for short times and the gas concentration.

## 5. Summary and Conclusions

A phenomenological description was developed for multi-gas diffusion into a closed chamber coated with a planar gas-selective membrane. This description allows for exact calculations of both the concentration and pressure within the membrane and measurement chamber of a sensor cell that forms the basis of membrane-based gas sensor technology. The boundary conditions that were examined represent two operating modes for measurement: case (I) starts from an established dynamic equilibrium, *i.e.*, maximum gas flow through the gas-selective membrane, and case (II) starts from thermodynamic equilibrium (no gas flow). The validity of different approximate solutions vital to realizing the measurements was discussed with respect to the exact theory.

Normalization of the pressure response from one measurement chamber to the response of a second measurement chamber, which is expressed by Equation (10), forms the theoretical basis for identifying a gas component added to the gas matrix by a critical time. At this critical time, the answer function of that paired sensor cells intersects a separating function independent of the gas concentration. This general theoretical framework was transformed into an equivalent behavior of the simple pressure difference between the two measurement chambers. In this important special case, the gas can be identified via a single differential pressure sensor connected to both chambers. In addition, the concentration of the identified gas can be determined in this special case using the same differential pressure sensor. Thereby, depending on the cell geometry, the achievable accuracy is comparable to that for measurements with the corresponding single gas sensor cell.

Simulations for cells with varying geometries and different gas matrixes resulted in a range of critical times from 50 s to 850 s and demonstrated a large range for optimizing and adapting to particular measurement problems. For rapid gas discrimination, smaller cells with thinner membranes can be considered too. Due to their simple construction, such rapid gas sensors, operating, e.g., according to case (II), seem to be well suited for miniaturization/chip-based applications.

This study considered the gas components N_2_, O_2_, Ar, CH_4_, CO_2_, H_2_ and C_4_H_10_. Using measurement chambers coated with PDMS membranes, material data for the simulations were obtained from the literature. Both the membrane material and gases included in this study were considered as examples to introduce the measurement principle. Based on the given theory that sensor can be successfully designed using a high number of gas-selective membranes and can be applied to identify and quantify a high number of different gas components in various environments.

The theoretically postulated phenomenons were experimentally confirmed for various O_2_ and CO_2_ concentrations mixed with an air gas matrix. These experiments demonstrated both the concentration dependence of the signal excursion, which enables specific gas components to be quantified, and the concentration independence of the critical time, which allowed for identification.

## Figures and Tables

**Table 1.** PDMS material parameters for the computational simulations.

**Gas**	**P×10^6^**	**D×10^5^**	**S**
	**(cm^2^/s)**	**(cm^2^/s)**	**cm^3^(RTP)/(cm^3^** × **atm)**
CH_4_	7.24	1.27	0.57
H_2_	5.16	4.3	0.12
C_4_H_10_	375	2.5	15
Ar	4.62	1.4	0.33
CO_2_	24.2	1.1	2.2
O_2_	4.96	1.6	0.31
N_2_	2.25	1.5	0.15

RTP—Room Temperature and Pressure (20 °C, 1 atm).

**Table 2.** Numerical eigenvalues and coefficients, $\beta_{i}^{j}$, for Equation (6) for cell I (*AL*/*V* ≪ 1).

**Gas**	*λ*_1_	$\beta_{i=1}^{j}$		*λ*_2_	$\beta_{i=2}^{j}$	

**“j”**		**Case (I)**	**Case (II)**		**Case (I)**	**Case (II)**
CH_4_	0.237	1.000	1.009	3.160	6.481 × 10^−5^	−1.135 × 10^−2^
H_2_	0.109	1.000	1.002	3.145	2.939 × 10^−6^	−2.423 × 10^−3^
C_4_H_10_	0.988	0.975	1.154	3.542	2.201 × 10^−2^	−1.999 × 10^−1^
Ar	0.181	1.000	1.005	3.152	2.199 × 10^−5^	−6.620 × 10^−3^
CO_2_	0.453	0.999	1.034	3.210	8.885 × 10^−4^	−4.171 × 10^−2^
O_2_	0.175	1.000	1.005	3.151	1.942 × 10^−5^	−6.223 × 10^−3^
N_2_	0.122	1.000	1.003	3.146	4.585 × 10^−6^	−3.026 × 10^−3^

**Table 3.** Numerical eigenvalues and coefficients, $\beta_{i}^{j}$, for Equation (6) for cell II (*AL*/*V* = 1).

**Gas**	*λ*_1_	$\beta_{i=1}^{j}$		*λ*_2_	$\beta_{i=2}^{j}$	

**“j”**		**Case (I)**	**Case (II)**		**Case (I)**	**Case (II)**
CH_4_	0.690	0.995	1.078	3.312	4.993 × 10^−3^	−9.750 × 10^−2^
H_2_	0.340	1.000	1.019	3.179	2.782 × 10^−4^	−2.345 × 10^−2^
C_4_H_10_	1.473	0.857	1.268	4.425	8.851 × 10^−2^	−4.084 × 10^−1^
Ar	0.545	0.998	1.049	3.243	1.890 × 10^−3^	−6.055 × 10^−2^
CO_2_	1.105	0.959	1.186	3.68	3.472 × 10^−2^	−2.490 × 10^−1^
O_2_	0.530	0.998	1.046	3.237	1.685 × 10^−3^	−5.722 × 10^−2^
N_2_	0.378	1.000	1.024	3.189	4.281 × 10^−4^	−2.905 × 10^−2^

**Table 4.** Numerical eigenvalues and coefficients, $\beta_{i}^{j}$ , for Equation (6) for cell III (*AL*/*V* ≫ 1).

**Gas**	*λ*_1_	$\beta_{i=1}^{j}$		*λ*_2_	$\beta_{i=2}^{j}$	

**“j”**		**Case (I)**	**Case (II)**		**Case (I)**	**Case (II)**
CH_4_	1.340	0.905	1.246	4.090	7.073 × 10^−2^	−3.561 × 10^−1^
H_2_	0.918	0.982	1.134	3.474	1.622 × 10^−2^	−1.726 × 10^−1^
C_4_H_10_	1.560	0.816	1.273	4.681	9.058 × 10^−2^	−4.242 × 10^−1^
Ar	1.217	0.938	1.217	3.850	5.064 × 10^−2^	−2.996 × 10^−1^
CO_2_	1.503	0.844	1.271	4.510	9.041 × 10^−2^	−4.163 × 10^−1^
O_2_	1.201	0.941	1.212	3.823	4.813 × 10^−2^	−2.921 × 10^−1^
N_2_	0.988	0.975	1.154	3.542	2.201 × 10^−2^	−1.999 × 10^−1^

**Table 5.** Simulated cell configurations and gas matrix compositions.

**Case**	**Cell I**		**Cell II**		**Gas Matrix**

	*L*_1_**(mm)**	*V*_1_/*A*_1_**(mm)**	*L*_2_**(mm)**	*V*_2_/*A*_2_**(mm)**	
I, II	0.1	1	1	1	air
Ia, IIa	0.1	0.1	1	1	air
Ib, IIb	0.1	0.1	0.1	1	air
Ic, IIc	0.1	1	1	1	Ar
